# Genomic Characterization of a Novel Freshwater Cyanophage Reveals a New Lineage of Cyanopodovirus

**DOI:** 10.3389/fmicb.2021.768868

**Published:** 2022-01-12

**Authors:** Dong Zhang, Yiliang He, Karina Yew-Hoong Gin

**Affiliations:** ^1^NUS Environmental Research Institute (E2S2-CREATE), National University of Singapore, Singapore, Singapore; ^2^School of Environmental Science and Engineering, Shanghai Jiao Tong University, Shanghai, China; ^3^Department of Civil and Environmental Engineering, National University of Singapore, Singapore, Singapore

**Keywords:** cyanophage, phylogenetic analysis, metagenomic mapping, new evolutionary lineage, freshwater *Synechococcus*, tropical freshwater environments

## Abstract

Cyanobacteria are one of the dominant autotrophs in tropical freshwater communities, yet phages infecting them remain poorly characterized. Here we present the characterization of cyanophage S-SRP02, isolated from a tropical freshwater lake in Singapore, which infects *Synechococcus* sp. Strain SR-C1 isolated from the same lake. S-SRP02 represents a new evolutionary lineage of cyanophage. Out of 47 open reading frames (ORFs), only 20 ORFs share homology with genes encoding proteins of known function. There is lack of auxiliary metabolic genes which was commonly found as core genes in marine cyanopodoviruses. S-SRP02 also harbors unique structural genes highly divergent from other cultured phages. Phylogenetic analysis and viral proteomic tree further demonstrate the divergence of S-SRP02 from other sequenced phage isolates. Nonetheless, S-SRP02 shares synteny with phage genes of uncultured phages obtained from the Mediterranean Sea deep chlorophyll maximum fosmids, indicating the ecological importance of S-SRP02 and its related viruses. This is further supported by metagenomic mapping of environmental viral metagenomic reads onto the S-SRP02 genome.

## Introduction

Cyanobacteria are the dominant autotrophs in freshwater and marine environment (Agawin et al., 2003; Saad and Atia, 2014). In particular, the cyanobacteria *Prochlorococcus* and *Synechococcus*, are major components of the cyanobacteria community across diverse environments, contributing up to 98% of the chlorophyll-*a* biomass (Scanlan and West, 2002; Jakubowska and Szela̧g-Wasielewska, 2015). Coexisting with cyanobacteria, cyanophages are widespread biological entities playing key roles in the ecological dynamics and biological diversity of cyanobacteria (Suttle, 2000; Sullivan et al., 2003). Furthermore, cyanophage infection mediates biogeochemical cycling of nutrients through lytic activity as well as cyanophage reprogramming host metabolic activity (Zimmerman et al., 2020).

Marine cyanophages have been widely studied and characterized, especially those infecting *Synechococcus* and *Prochlorococcus* (Dekel-Bird et al., 2013; Huang et al., 2015b; Zimmerman et al., 2020). In total, over 120 genomes of cyanophages have been sequenced and deposited in the Virus host database (accessed 10th March 2021). However, only a small portion of cyanophage genome belong to freshwater cyanophages. In 2007, complete genome of the first freshwater cyanopodovirus Pf-WMP4 was reported (Liu et al., 2007). The first freshwater cyanophage Ma-LMM01 infecting *Microcystis aeruginosa* was isolated in 2006 with its complete sequence reported in 2008 (Yoshida et al., 2008). The first tailless freshwater cyanophage PaV-LD was isolated in 2009 and sequenced in 2012 (Gao et al., 2012). In 2015, a new lineage of cyanophage is revealed by the isolation of S-EIV1 infecting polar freshwater *Synechococcus* (Chenard et al., 2015). The first cyanophage infecting freshwater *Pseudanabaena* was reported in 2020 (Zhang et al., 2020). It is the second tail-less cyanophage isolated and harbors substantial proportion of genes with unknown function. Despite limited studies, discovery on freshwater cyanophage keep revealing novel cyanophages of distinct genomic content and freshwater cyanophages tend to have distinct genomic content from their marine counterpart. To date, only 18 genomes of freshwater cyanophages infecting various host cyanobacteria have been sequenced. Based on morphology, 6 myoviruses, 5 podoviruses, 4 siphoviruses, and 2 tail-less phages have been isolated from freshwater environments and sequenced (Morimoto et al., 2020; Yang et al., 2020). Of these, S-EIV1, S-CRM01, S-SRP01, S-SRM01, S-LBS01, and S-2L were found to infect freshwater *Synechococcus* spp. (Dreher et al., 2011; Chenard et al., 2015; Zhong et al., 2018; Zhang et al., 2021). Interestingly, S-CRM01 was isolated from freshwater environment but shares close phylogenetic relationship with marine cyanomyoviruses (Dreher et al., 2011). This relatedness between freshwater and marine cyanophages is further supported by the isolation of S-SRM01 and S-SRP01 which shared unprecedented genetic similarity with their marine counterpart (Zhang et al., 2021).

Here we report for the first time, the isolation and characterization of a novel freshwater cyanophage S-SRP02 infecting *Synechococcus* sp. Strain SR-C1. Both the host and phage were isolated from a tropical urban freshwater (Gin et al., 2021). Unlike the previous discovery of S-SRP01 and S-CRM01, S-SRP02 does not cluster with any established phylogenetic groups of cyanoviruses infecting *Synechococcus* or *Prochlorococcus*. This phage is a distinct new member in freshwater cyanophages that infect *Synechococcus*, an often-dominant cyanobacterium in tropical waters. Furthermore, recent studies have highlighted the novel ability of freshwater *Synechococcus*, including SR-C1, to produce cylindrospermopsin toxin (Gin et al., 2021; Tavakoli et al., 2021). Our work on S-SRP02 not only expands the current understanding of freshwater cyanophage diversity and its control of potentially toxic *Synechococcus* populations, but also highlights its ecological prevalence through identifying S-SRP02 related viruses in distant environments elsewhere.

## Materials and Methods

### Host Cells

The *Synechococcus* sp. strain SR-C1 was isolated as described previously (Zhang et al., 2020). The strains were isolated by micro-pipetting from a freshwater lake sample into sterile MLA medium (Bolch and Blackburn, 1996) at 25°C. Identification of the strains was determined through whole genome sequencing (Gin et al., 2021). The cultures were then incubated and maintained in batch culture at 25°C under low irradiance (20 μmol photons m^–2^s^–1^) with a 12-h/12-h light/dark cycle.

### Cyanophage Isolation

Cyanophage S-SRP02 were isolated from viral concentrates collected from freshwater lake as described above. Briefly, 450 ml of sample water was filtered through 0.2 μm (Nucleopore) pore size filters and then concentrated through ultrafiltration at 5,000 *g* for 15 min with centrifugal unit cut-off of 100-KDa-MW (Amicon^®^ Ultra-15 Centrifugal Filter Units; Millipore). Viral concentrates were serial-diluted before adding to exponentially growing cultures of host cyanobacteria in a 96-well plate and incubating at 28°C under low irradiance (20 μmol photons m^–2^s^–1^) with a 12-h/12-h light/dark cycle for 14 days. A more than 50% decrease in host population, as measured by flow cytometry using a CytoFLEX flow cytometer (Beckman Coulter Inc., Brea, CA, United States), indicated culture lysis. The discriminator was set on Forward scatter height (FSC-H) and Allophycocyanin height (APC-H), and samples (1 mL) were analyzed at a rate of 30 μL/min. 1.0 mm FluoSpheres microsphere beads (Thermo Fisher Scientific Inc., United States) were added for absolute counting. After three rounds of extinction dilution (Nagasaki and Yamaguchi, 1997) in 96-well microtiter plates, a clonal cyanophage isolate was obtained in the well of highest dilution rate.

### Cyanophage Amplification and Purification

300 μl of the virus isolate was added to 30 ml cultures of host cyanobacteria to produce enough phage progeny for subsequent analysis. Upon lysis, the lysates were centrifuged at 15,000 × *g* for 5 min and the supernatant containing the majority of viral particles was filtered through a 0.22 μm syringe filter (Minisart Syringe Filter, Sartorius) to ensure that the filtrate was free of host cell contamination.

### One-Step Growth Curve

To examine the adsorption profile as well as infection process of S-SRP02 on *Synechococcus* SR-C1, one-step growth curve was performed as previously described (Zhang et al., 2021). Briefly, in biological triplicates, purified phage was added to exponentially growing cultures of *Synechococcus* SR-C1 at MOI of ∼2 and low initial host cell concentration of 9.5 × 10^4^ cells/mL to avoid re-infection during the experiment, equal volume of MLA medium was added to serve as control. 1 mL samples were taken at every 3 h throughout a duration of 48 h after phage inoculation and filtered through 0.2 μm PC membrane (Isopore; Millipore) with the membrane being washed three times with MLA medium to minimize free phage trapped on the membrane. 200 μL of filtrate was used for viral DNA extraction and subsequently quantified using qPCR with new primers listed here (Supplementary Table 1). The primer pair was designed by uploading major capsid protein gene sequences of S-SRP02 onto Primer-BLAST as PCR template and default primer parameters were chosen. Primer specificity checking was also performed against the nr database to ensure that the primers are specific to the template and no other sequences in the nr database could be amplified by the primer sets (Ye et al., 2012). The 20 μL qPCR reaction mix consist of 10 μL of FastStart Universal Probe Master (Rox), 1 μM of each primer, 3 μL of nuclease free water and 5 μL of DNA template. Thermal cycling was conducted in a StepOnePlus™ Real-Time PCR System (Applied Biosystems) with the following program: 10 min denaturation at 95°C, followed by 40 cycles of denaturation at 95°C for 30 s, annealing at 58°C for 30 s and extension at 72°C for 30 s. To test whether the free phage abundance quantified by qPCR consist of unpackaged phage DNA, free phage samples taken at each time interval are digested with DNase I. qPCR quantification of free S-SRP02 in digested samples are conducted same as described above.

### Transmission Electron Microscopy

Purified lysate was used as sample for TEM imaging. For staining, 20 μl of gadolinium triacetate (1% w/w) was adsorbed to the surface of copper grids at room temperature for 1 min. Excess liquid was blotted off from the side of copper grid with clean filter paper. The grids were viewed and photographed on a JEOL JEM-2100F field emission gun transmission electron microscope.

### Host Range

Cyanobacteria isolates from local freshwaters and overseas culture collections were used to test the host range of S-SRP02 (Table 1). 20 μl of phage lysate was added to cultures of the exponentially growing cyanobacteria listed in 96 well plates. For each cyanobacteria strain tested, one well was inoculated with 0.02 ml of MLA medium to serve as a control while 6 wells were inoculated with phage lysate. A 50% decline in OD reading as compared to the control indicates infectivity (Yeo and Gin, 2014).

**TABLE 1:** List of Cyanobacteria used for host range test.

Species	Strain	Origin	Susceptibility to S-SRP02
*M. aeruginosa*	CS569	CSIRO culture collection	–
*A. cylindrica*	CS172	CSIRO culture collection	–
*Cylindrospermopsis*	CS511	CSIRO culture collection	–
*A. circinalis*	CS337	CSIRO culture collection	–
*Synechococcus*	SR-C1	Singapore freshwater lake	+
*Synechococcus*	SR-C21	Singapore freshwater lake	–
*Synechococcus*	SR-C6	Singapore freshwater lake	–
*Synechococcus*	SR-R4S1	Singapore freshwater lake	–
*Microcystis*	SR-I31	Singapore freshwater lake	–
*Microcystis*	SR-I1	Singapore freshwater lake	–
*Limnothrix*	SR-Fila1	Singapore freshwater lake	–
*Pseudanabaena*	SR-C8	Singapore freshwater lake	–
*Cylindrospermopsis*	CY2.2	Singapore freshwater lake	–
*Pseudanabaena*	M6A	Singapore freshwater lake	–
*Nostoc punctiforme*	ATCC 29133	American Type Culture Collection	–

### DNA Extraction, Purification, and Sequencing

Host cyanobacteria were grown in 25 ml of MLA media at 25°C under low irradiance (20 μmol photons m^–2^s^–1^) with a 12-h/12-h light/dark cycle until lysis. The lysates were purified as described above. To remove free nucleic acid, the lysate was treated with DNase I and subsequently concentrated with 100-kDa-MW cut-off ultrafiltration centrifugal tubes (Amicon^®^ Ultra-15 Centrifugal Filter Units; Millipore) at 4,000 × *g* for 15 min to a final volume of 0.2 ml. QIAamp DNA Mini Kit was used to extract viral DNA with 5 μl of RNase A added in the first step to remove free RNA. The cyanophage genome was sequenced using an Illumina High throughput sequencer, with a 150-bp paired-end library constructed using a NEB Next Ultra DNA Library Prep Kit.

### Genome Assembly

The sequencing data were trimmed using BBDuk (version 38.18) to remove adaptors and Phix reads. Reads were *de novo* assembled into contigs by MetaSPAdes version 3.13.0 (Nurk et al., 2017). The whole genome sequence of the phage has been submitted to GenBank under accession MW822601.

### Genome Annotation

The open reading frames (ORFs) were predicted using Prodigal (V2.6.3) in meta mode (Hyatt et al., 2010). Homology searching was performed with Diamond (V0.9.14.115) (Buchfink et al., 2015) against the NCBI non-redundant (nr) database (accessed in July 2020). Sequences with *e*-values < 10^–5^ were considered to be homologs. HHpred analysis against the protein data bank (PDB) and Pfam database were used to predict more distant homologs (Soding et al., 2005). S-SRP02 genome was uploaded to tRNAscan-SE 2.0 server for identification of cyanophage encoded tRNA (Lowe and Chan, 2016).

### Phylogenetic Analysis

For phylogenetic analysis of S-SRP02, concatenation of the terminase, DNA polymerase and major capsid protein genes were compared phylogenetically with those from other cyanopodoviruses as well as uncultured viruses from Mediterranean sea deep chlorophyll maximum metagenomic fosmids (Table 2) using Mega-X software (version 10.1.6) (Kumar et al., 2018). ClustalX was used to align the inferred amino-acid sequences with default parameters (Larkin et al., 2007). The Jones-Taylor-Thornton (JTT) model was selected and maximum likelihood tree was constructed with 100 bootstrap replicates (Jones et al., 1992). ViPTree was used to construct the virus proteomic tree comparing S-SRP02 and 2620 dsDNA phages deposited in the Virus-Host Database^1^ (Nishimura et al., 2017).

**TABLE 2:** List of phages used for phylogenetic analysis.

Accession	Organism
NC_024358.1	Anabaena phage A-4L
NC_020865.1	Cyanophage KBS-P-1A
NC_022751.1	Cyanophage PP
NC_016656.1	Cyanophage P-SSP2
NC_020872.1	Cyanophage SS120-1
NC_009531.1	Cyanophage Syn5
NC_009551.1	Phormidium phage Pf-WMP3
NC_008367.1	Phormidium phage Pf-WMP4
NC_020878.1	*Prochlorococcus* phage P-GSP1
HQ332139.1	*Prochlorococcus* phage P-RSP2
NC_020835.1	*Prochlorococcus* phage P-SSP10
NC_020874.1	*Prochlorococcus* phage P-SSP3
NC_006882.2	*Prochlorococcus* phage P-SSP7
NC_003390.2	*Synechococcus* phage P60
NC_025456.1	*Synechococcus* phage S-CBP1
NC_025455.1	*Synechococcus* phage S-CBP2
NC_025461.1	*Synechococcus* phage S-CBP3
NC_025464.1	*Synechococcus* phage S-CBP4
KJ410740.1	*Synechococcus* phage S-EIVl
NC_020867.1	*Synechococcus* phage S-RIP1
NC_020838.1	*Synechococcus* phage S-RIP2
MW015080	*Synechococcus* phage S-SRP01
NC_047700.1	Uncultured phage_MedDCM-OCT-S37-C6
NC_047706.1	Uncultured phage_MedDCM-OCT-S31-C1
NC_047707.1	Uncultured phage_MedDCM-OCT-S38-C3

### Recruitment of Metagenomic Reads to S-SRP02 Genome

To explore the presence of viral sequences similar to S-SRP02 in aquatic environments, viral metagenomic data were recruited onto the S-SRP02 genome (Zhao et al., 2013). Metagenomic data (Supplementary Table 2; Roux et al., 2012; Brum et al., 2015; Arkhipova et al., 2018; Ruiz-Perez et al., 2019; Moon et al., 2020) were first made into a BLAST nucleotide database and queried with the predicted protein sequences of S-SRP02 using tBLASTn (*E*-values < 10^–5^). Metagenomic reads with BLAST-hit to S-SRP02 were then extracted based on its sequence header ID. The reads extracted were used as query to BLASTx (*E*-values < 10^–5^, max_target_seqs = 1) against viral protein sequences containing the predicted protein sequences of S-SRP02 phage listed in Table 3 and the other 3683 phage genomes of the NCBI Reference Sequence database (RefSeq, accessed on 17th March 2021). If the best hit was related to S-SRP02 instead of other phages, it was recruited as viral sequences similar to S-SRP02 and mapped onto S-SRP02 genome using ggplot2 (Wickham, 2009). The total number of blast hits to the S-SRP02 was normalized by dividing by the length of the S-SRP02 genome (in kb) and the size of metagenome data used (in Gb). Similar recruitment analysis was performed for the other phage genomes listed in Table 3. In order to avoid underestimating the relative abundance of cyanophages with similar gene content, phage genomes with genetic proximity were grouped and their relative abundance was summed to represent the phage group.

**TABLE 3:** List of viral genomes used for metagenomic recruitment analysis.

Phage or phage group (based on genetic proximity)	Genome size	Accession number
S-SRM01	240,842	MW015081
S-CRM01	178,563	NC_15569
S-EVI1	79,178	KJ410740
P-SSP10	47,325	NC_20835
P-RSP2	42,257	HQ332139
Pf-WMP4	40,938	NC_008367
A-4L	41,750	NC_024358
S-SRP02	42,134	MW822601
**MPP-A**		
P60	46,675	NC_003390
S-CBP2	46,237	NC_025455
S-CBP42	46,237	NC_029031
SYN5	46,214	NC_009531
**MPP-B2**		
P-GSP1	44,945	NC_020878
P-SSP2	45,890	NC_016656
P-SSP3	46,198	NC_020874
P-SSP7	45,176	NC_006882
**MPP-B3**		
KBS-P-1A	45,730	NC_020865
S-RIP1	44,892	NC_020867.
S-RIP2	45,782	NC_020838
**MPP-B4**		
S-CBP1	46,547	NC_025456
S-CBP3	45,871	NC_025461
S-CBP4	44,147	NC_025464
S-SRP01	45,017	MW015080
**Pf-WMP3 LIKE**		
Pf-WMP3	43,249	NC_009551
PP	42,480	NC_022751

## Results and Discussion

### Morphology

**FIGURE 1:**
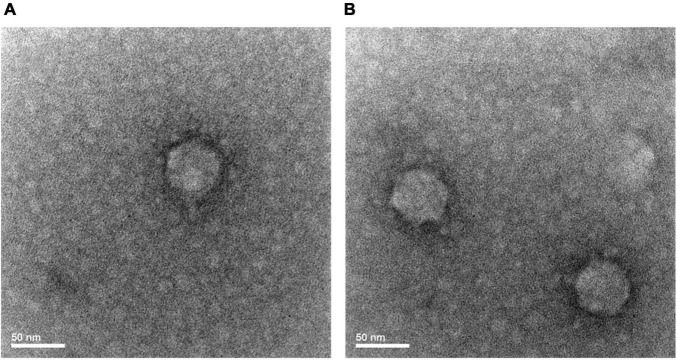
Transmission electron microscope image of S-RP02 **(A,B)**. Short tail is only visible under certain phage particle orientation **(A)**.

S-SRP02 is a short-tail virus with a capsid diameter of approximately 50 nm (Figure 1). Similar capsid size and shape is shared among S-SRP02 and other podoviruses infecting *Synechococcus* spp., suggesting conservation in the structural proteins of cyanopodoviruses (Huang et al., 2015a).

### Host Specificity and Growth Curve

Infectivity of S-SRP02 was tested against local freshwater cyanobacteria isolates as well as cyanobacteria cultures obtained from overseas culture collections. The host range test (Table 1) revealed that S-SRP02 has a narrow host range as it does not infect cyanobacteria of other species nor other *Synechococcus* sp. isolated from the same lake as its host. The absence of cyanophage encoded tRNA genes in S-SRP02 genome may have limited its host range. The codon usage of bacteriophage was shown to be strongly adapted to their specific host but different from non-host (Bahir et al., 2009). Cyanophage tRNA could contribute substantially to the translation efficiency of cyanophage genes, which is especially important for successful infection when the host has differing codon usage pattern (Enav et al., 2012; Xu et al., 2018).

**FIGURE 2:**
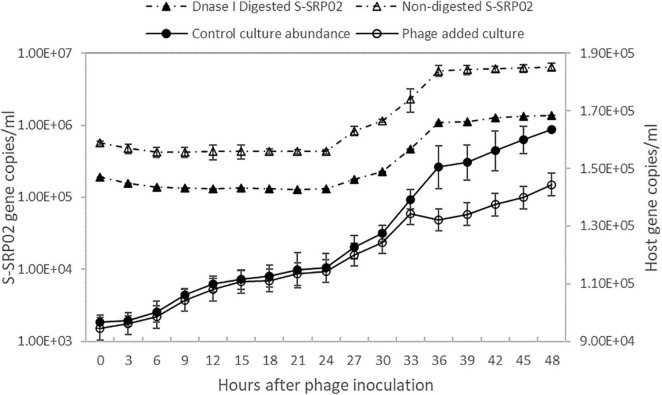
The growth of host *Synechococcus* sp. strain SR-C1 is shown above. Host cyanobacteria concentration is measured by flow cytometry. Open circle reflects host concentration of culture inoculated with S-SRP02 and dark dot reflects host concentration of control culture. Free S-SRP02 phage abundance is denoted by open triangle and dark triangle represent abundance of S-SRP02 after DNase I digestion.

As shown in Figure 2, free S-SRP02 abundance decreased from 0 to 6 h post inoculation., S-SRP02 abundance started to increase from 24 h post inoculation to 36 h post inoculation, indicating a latent period of approximately 24 h. This is a relatively long latent period as compared to marine cyanopodoviruses infecting picocyanobacteria, which typically range from 1 h (Syn5 phage) to ∼9 h (S-CBP2) (Pope et al., 2007; Wang and Chen, 2008). At the other end of the spectrum of cyanophages infecting picocyanobacteria, S-EIV1 has a much longer latent period of more than 7 days (Chenard et al., 2015). Interestingly, the production of S-SRP02 phage was only observed during illumination period from 24 to 36 h. Based on the infection profile, the burst size of S-SRP02 is estimated to be ∼250. Such a high burst size is unusual as compared to burst size of cyanopodovirus of similar genome size, which normally range between 50 and 120 (Sullivan et al., 2005; Wang and Chen, 2008; Zhou et al., 2013; Ou et al., 2015). This difference might arise from the different quantification method used. Previous studies mostly used plaque assay for phage quantification, quantifying only infective phages. However, using qPCR both infective and non-infective phages can be quantified. The difference may arise due to the release of unpackaged phage DNA. Studies have shown that cyanophage infection appears to be energy limited for protein synthesis instead of DNA (Mahmoudabadi et al., 2017; Puxty et al., 2018), making it a reasonable assumption that bacteria lysis might be accompanied by release of unpackaged phage DNA which could possibly arise due to the lack of phage protein production or inefficient morphogenesis. To test for this hypothesis, the free phage samples at each time point were subjected to DNase I digestion to remove unpackaged viral nucleic acid. Free phage abundance in the digested samples were quantified using qPCR. Surprisingly, a significant proportion of phage gene copy detected was from unpackaged phage DNA as shown in Figure 2. On average, 29% of the phage gene copy number was attributed to packaged phage DNA, demonstrating that majority of phage gene copy number measured by qPCR is free viral nucleic acid instead of DNA encapsulated in phage protein. This observation agrees with the previous studies that cyanophage infection is energy limited in protein synthesis instead of DNA replication. It also demonstrates the importance of DNase digestion in determining phage titer using qPCR. With free S-SRP02 abundance measured from DNase I digested sample, burst size of S-SRP02 is estimated to be 50. This is comparable to the normal burst size range of cyanophages measured by plaque assay.

### Genomic Characteristics

**FIGURE 3:**
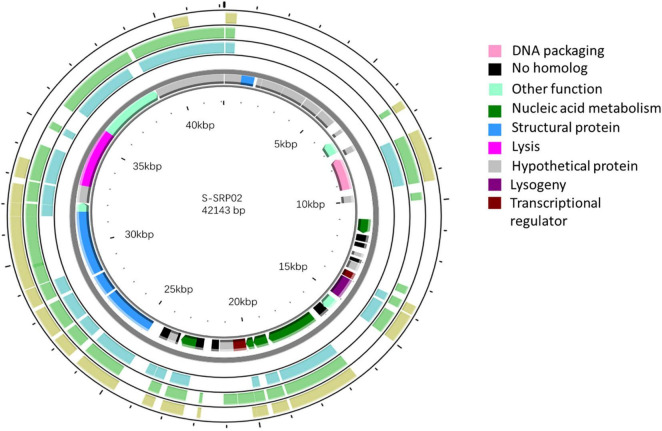
Genomic map of S-SRP02. Circles from innermost to outermost ring correspond to predicted ORFs (BLASTp, nr database, *E*-value < 0.00001) on (i) reverse strand and (ii) forward strand, (iii) tBLASTx hits (*E*-value < 0.00001) of S-SRP02 genome against uncultured phage MedDCM-OCT-S37-C6, (iv) MedDCM-OCT-S38-C3 and (v) MedDCM-OCT-S31-C1. Legends on the right indicate predicted functions of S-SRP02 ORFs on the two inner rings (i) and (ii).

S-SRP02 is a double stranded DNA virus with genome size of 42,143 and average GC content of 63.4%. Most ORFs in S-SRP02 could not be assigned putative function based on functional annotation against the NCBI nr database. Out of 47 ORFs predicted, 20 ORFs have significant similarities to other genes of known functions (BLASTp, *e*-value cutoff of 10^–5^) associated with structural proteins, nucleic acid metabolism, DNA packaging, lysis, lysogeny, transcriptional regulator and other functions (Figure 3). We were unable to identify distant homologs for further annotation of S-SRP02 genes using HHpred analysis. In total, 28 ORFs have no similarity to known viral genes, including those with unknown function.

### Auxiliary Metabolic Genes

Host-derived auxiliary metabolic genes are widely present in the cyanophage genome playing crucial roles in cyanophage-host interaction and infection cycle (Gao et al., 2016; Zimmerman et al., 2020). Six host-derived genes were found to be core genes in marine cyanopodovirus pan-genome (Huang et al., 2015a). They are involved in various metabolic functions such as photosynthesis (*psbA*, hli), DNA biosynthesis (*nrdA/nrdB*, *thyX*), programmed cell death (*mazG*) and carbon metabolism (*talC*). Interestingly, no host derived genes could be identified in S-SRP02 genome (Table 4), indicating that S-SRP02 is evolutionarily distant from marine cyanopodoviruses. Cyanophages containing *psbA* genes were found to upregulate cellular photosynthetic activities in both the phage isolate and environmental samples (Puxty et al., 2018; Sieradzki et al., 2019). Given the relatively long latent period of S-SRP02, it is likely there are novel host-derived auxiliary metabolic genes present which help to provide energy and nutrients for phage progeny production during lytic infection. Further study is needed to better explain the lack of host-derived auxiliary metabolic genes in S-SRP02.

**TABLE 4:** Predicted ORFs of cyanophage S-SRP02 with similarity to genes of known function.

ORF	GenBank ID	% identity	*E*-value	Putative protein encoded	Organism
2	BAR21487.1	52	1.10E-19	Phage tail fiber protein	Uncultured Mediterranean phage uvMED
10	MBD2422814.1	60.3	7.60E-78	C39 family peptidase	*Cyanobium* sp. FACHB-13342
12	NBW77013.1	70	2.60E-252	DNA maturase B	Sphingomonadaceae bacterium
14	BAR30560.1	29.4	5.60E-17	DNA polymerase III beta subunit	Uncultured Mediterranean phage uvMED
20	NBV61882.1	69.5	2.10E-32	MarR family transcriptional regulator	Rhodobacteraceae bacterium
21	NBV61881.1	74.7	2.60E-145	Site-specific integrase	Rhodobacteraceae bacterium
22	NDC35227.1	68.6	4.00E-27	KilA-N domain-containing protein	Synechococcaceae bacterium WB9_2_112
25	YP_009777926.1	40.5	8.40E-166	DNA-directed RNA polymerase	Uncultured phage_MedDCM-OCT-S38-C3
26	YP_009777617.1	33.7	5.00E-15	Phage single-stranded DNA-binding protein	Uncultured phage_MedDCM-OCT-S37-C6
27	NBW76986.1	79.3	5.00E-49	RusA family crossover junction endodeoxyribonuclease	Sphingomonadaceae bacterium
28	YP_009777929.1	46.9	6.90E-40	Transcriptional regulator NrdR	Uncultured phage_MedDCM-OCT-S38-C3
34	YP_009777611.1	49.2	5.50E-63	DNA polymerase I	Uncultured phage_MedDCM-OCT-S37-C6
38	NBW76996.1	74.9	1.30E-210	Head-to-tail connector (portal) protein	Sphingomonadaceae bacterium
39	YP_009777939.1	37.2	1.10E-38	Capsid assembly protein	Uncultured phage_MedDCM-OCT-S38-C3
40	YP_009777898.1	55.7	1.30E-94	Major capsid protein	Uncultured phage_MedDCM-OCT-S31-C1
41	YP_009777638.1	64.6	9.40E-75	Tail tubular protein A	Uncultured phage_MedDCM-OCT-S37-C6
42	BAR25425.1	67.4	0.00E + 00	Putative tail tubular protein B	Uncultured Mediterranean phage uvMED
43	YP_009777594.1	43.3	2.50E-26	Putative acetyltransferase	Uncultured phage_MedDCM-OCT-S37-C6
45	MBD1877251.1	51	1.80E-29	N-acetylmuramoyl-L-alanine amidase	*Nodosilinea* sp. FACHB-131
46	BAR25421.1	39	3.00E-160	Chromosome segregation ATPase-like protein	Uncultured Mediterranean phage uvMED

### Lysis Gene

Lysozyme, responsible for lysing cyanobacteria cell walls, is commonly found in cyanophages infecting various cyanobacteria from diverse environments (Yoshida et al., 2008; Chenard et al., 2015; Huang et al., 2015a). However, there is a lack of lysozyme homolog identified in the S-SRP02 genome. Instead, ORF45 encoding for putative N-acetylmuramoyl-L-alanine amidase (MurNAc-LAA) was found through gene annotation. MurNAc-LAAs are responsible for cleaving glycosidic bonds in peptidoglycan in a different manner from lysozyme (Vollmer et al., 2008). MurNAc-LAA is likely to be the gene involved in lysing the host cell wall, suggesting that S-SRP02 adopts a unique lysis strategy.

### Structural Gene

Sharing a common set of structural genes is one key criterion used for taxonomic classification of viruses (Lavigne et al., 2008, 2009). Six structural genes were identified in the S-SRP02 genome. A majority of these genes shared highest sequence similarity with structural genes found in uncultured phages assembled from the Mediterranean sea deep chlorophyll maximum metagenomic fosmids (Mizuno et al., 2013). S-SRP02 structural genes are highly divergent from cultured cyanopodoviruses discovered previously. With an *e*-value cutoff of <10^–5^, no homologs could be identified between structural genes found in S-SRP02 and those from cyanopodoviruses belonging to either the MPP-A or MPP-B clade (Labrie et al., 2013; Huang et al., 2015a). Furthermore, no homologs from cultured phage were identified with ORF40 encoding for putative major capsid protein. This finding is similar to the novel lineage of myovirus represented by S-TIM5, which lacks homologs of structural in any known phage (Sabehi et al., 2012). This suggests that S-SRP02 forms a novel evolutionary lineage of cyanopodovirus with unique set of structural genes.

### Phylogenetic Analysis

**FIGURE 4:**
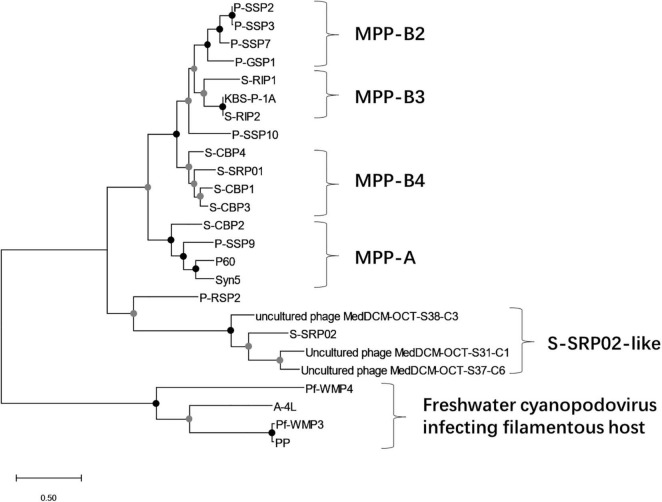
Maximum likelihood tree of inferred amino acid based on concatenated genes (terminase, DNA polymerase, and major capsid protein). Bootstrap values are indicated as black (100%) and gray (75–99%) at the nodes (100 replicates).

To have a comprehensive understanding of the evolutionary relationship between S-SRP02 and its related phages, a phylogenetic tree of concatenated genes including terminase (terL), DNA polymerase and major capsid protein was constructed (Figure 4). As mentioned above, S-SRP02 clearly did not cluster within the MPP-A nor MPP-B clade defined in previous research (Labrie et al., 2013; Huang et al., 2015a). S-SRP02 forms its own cluster with 3 uncultured phages as indicated by the S-SRP02-like cluster. Another cluster shown here is the cluster of freshwater cyanopodoviruses infecting filamentous cyanobacteria such as *Phormidium foveolarum* and *Dolichospermum* sp. This further suggests that S-SRP02 forms a novel evolutionary lineage of cyanophage.

**FIGURE 5:**
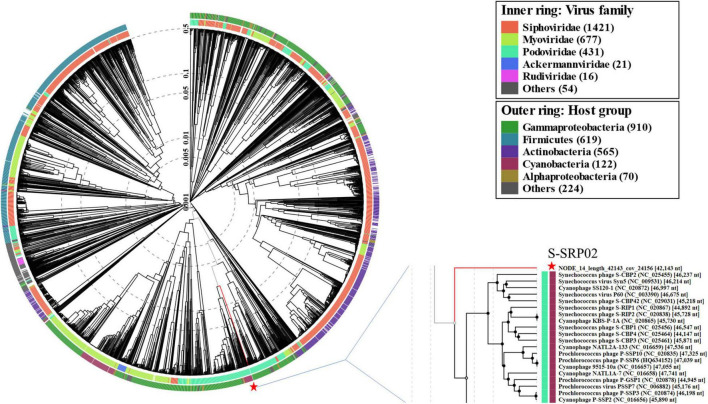
Proteomic tree constructed based on complete genome sequences of 2620 dsDNA phages deposited in Virus-Host Database (https://www.genome.jp/virushostdb/). S-SRP02 is highlighted with a red star.

To examine the evolutionary relationship of S-SRP02 from a wider perspective, a viral proteomic tree based on full genome sequences of all viruses and cyanophages deposited in the Virus-Host database was constructed using ViPTree (Figure 5). As indicated by the light blue color representing podovirus, S-SRP02 is evolutionarily closer to podovirus, agreeing with its morphological characteristics. Yet the evolutionary distance between S-SRP02 and other podoviruses remain large. Zooming into the adjacent section, the divergence between S-SRP02 and other cyanopodovirus is indicated by the branch length and lack of phage clustering with S-SRP02. This result further demonstrates that S-SRP02 is likely to represent a novel evolutionary lineage of cyanophage.

### S-SRP02 in the Environment

As shown in the previous annotation and phylogenetic relationship, S-SRP02 shares close proximity in gene content with 3 uncultured phages recovered from fosmid metagenomics in the Mediterranean deep chlorophyll maximum sample (Mizuno et al., 2013). A BLASTx analysis (*E*-value < 10^–5^) of S-SRP02 genome against sequences of MedDCM-OCT-S38-C3, MedDCM-OCT-S37-C6 and MedDCM-OCT-S31-C1 separately demonstrated gene synteny along a major part of the S-SRP02 genome. In total, 24 ORFs were shared between S-SRP02 and MedDCM-OCT-S38-C3, of which 13 ORFs encoded for putative proteins with known functions. Similarly, 19 ORFs were shared between S-SRP02 and MedDCM-OCT-S37-C6. 18 ORFs were shared between S-SRP02 and MedDCM-OCT-S31-C1 (Supplementary Table 3). The synteny between S-SRP02 and uncultured phages from MedDCM is visually represented in Figure 3. Putative proteins shared among S-SRP02 and all these fosmids encode for functions including terminase, tail fiber protein, head-tail connector, DNA-directed RNA polymerase, major capsid protein and capsid assembly protein transcriptional regulator (MarR). Phylogenetic affiliation of concatenated genes coupled with genetic similarities shared between the three fosmids and S-SRP02 indicates that these fosmids are from a relative of S-SRP02. Little sequence similarity is shared among S-SRP02 and other fosmids from the same study, suggesting that it actually represents an evolutionary group of viruses in the Mediterranean deep chlorophyll maximum (Mizuno et al., 2013).

### Metagenomic Recruitment Analysis

**FIGURE 6:**
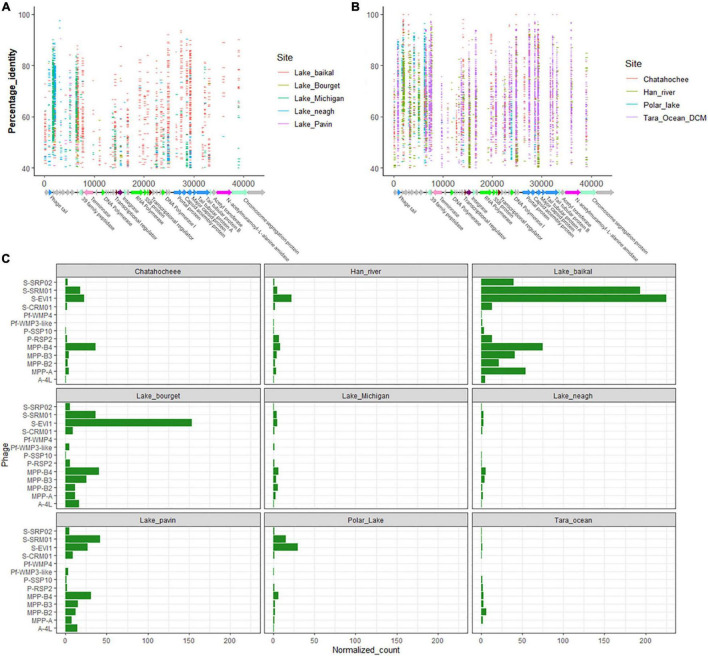
Prevalence of viral sequences similar to S-SRP02 in environmental viral metagenomic data. In **(A,B)**, each horizontal line represents a read recruited from one of the publicly available metagenomics data sets as indicated by the color legend on the right. Vertical axis corresponds to the percentage identity of S-SRP02 gene with predicted amino acid sequence of environmental viral raw reads. Horizontal axis corresponds to the position of read mapping on S-SRP02 genome. **(C)** Represents the relative abundance of various phage genomes from viral metagenomic data reflected by normalized recruited reads number.

To further understand the ecological prevalence and importance of S-SRP02 in the environment, metagenomic recruitments revealed that sequences similar to S-SRP02 genome are widespread across freshwater and marine environments. S-SRP02-like sequences are detected in all metagenomes (Figures 6A,B). Sequences similar to S-SRP02 genome were widely represented in Lake Baikal (Siberia), Chattahoochee River (North America) and Han River (South Korea) virome where sequence mapping could cover over 82% of genes predicted in S-SRP02. In Lake Neagh (Ireland) and Tara Ocean DCM (42 marine samples from 36 sampling sites across the globe), sequences similar to S-SRP02 are well represented since 65% of genes in S-SRP02 could be mapped in recruitment. This is not surprising since uncultured phages sharing close genetic characteristics with S-SRP02 were identified previously in the Mediterranean Sea Deep chlorophyll Maximum viral fosmids (Mizuno et al., 2013). In contrast, only a small portion (approximately 17%) of S-SRP02 genes could be mapped in recruitment against the Lake Bourget and Lake Pavin (France) metagenome. Despite poor representation in the two French lakes, sequences similar to the S-SRP02 genome are widely distributed in other metagenomes representing a variety of ecosystems across a wide geographical range. Overall, this indicates that S-SRP02 related viruses are prevalent in the aquatic ecosystem and are of ecological importance across both freshwater and marine environments.

Many genes of S-SRP02 are well represented in environmental metagenome. For example, structural genes clustered between 24 and 31 kbp (ORF38, ORF39, ORF40, and ORF42) are extensively mapped from Tara Ocean DCM and Lake Baikal (Figures 6A,B). 3,194 sequences were mapped to merely these 4 structural genes. These are also the few genes having the most number of recruited sequences from Tara Ocean DCM, indicating the ecological abundance of viral particles sharing similar structural genes with S-SRP02, suggesting that similar structural genes play important roles in the function and survival of phages similar to S-SRP02. The most extensively recruited S-SRP02 gene in Tara Ocean DCM is Terminase with 930 reads recruited. This suggests the prevalence of phage adopting similar DNA packaging strategies as S-SRP02. Interestingly, ORF45 encoding for N-acetylmuramoyl-L-alanine amidase is well represented in the Tara Ocean DCM sample with 514 reads recruited, suggesting the possible role of N-acetylmuramoyl-L-alanine amidase as a lysis gene in marine viruses related to S-SRP02 (Vollmer et al., 2008).

Other than functional genes, genes predicted to encode for hypothetical protein make up a major component of sequences similar to S-SRP02 in the environment. For example, in Tara Ocean DCM alone, 3,011 reads could be mapped to genes encoding for hypothetical protein. ORF44 and ORF29 encoding for hypothetical protein have 745 and 622 reads recruited, respectively, reminding us of the importance of genes with unknown function in the phage genome. Similarly, ORF4 encoding for hypothetical protein has 466 reads recruited from Lake Neagh and 479 reads recruited from Han River, making up 65% of the total reads recruited from Lake Neagh. The abundance of phage hypothetical genes, especially genes like ORF4, ORF29, and ORF44, occurring at high frequency in the environmental virome indicate their ecological importance in the dynamics and evolution of environmental phages similar to S-SRP02. Admittedly, there could be possible bacteria DNA contamination in viral metagenomes and this may interfere with the metagenomic mapping analysis. For example, ORF10 encoding for C39 family peptidase has the highest number of recruited reads in the Polar Lake metagenome and this could possibly be attributed to bacterial DNA contamination. However, ORF10 also has the second highest number of recruited reads in the Chattahoochee River metagenome which was considered to be free of bacteria DNA contamination based on the less than 0.02% bacteria 16 s content (Roux et al., 2013; Ruiz-Perez et al., 2019), indicating the potential role of C39 family peptidase as an important gene in the phage genome despite not being identified as a phage marker gene nor auxiliary metabolic gene.

The relative abundance of sequences similar to S-SRP02 displayed interesting results across different metagenomes (Figure 6C). High relative abundance is often attributed to S-SRM01, S-EIV1 and clusters of cyanopodovirus infecting picocyanobacteria. Sequences similar to S-SRP02 seem to be enriched in most of the metagenomes despite not being the dominant one. Higher S-SRP02 relative abundance is observed in freshwater metagenomes, consistent with the fact that S-SRP02 is a freshwater cyanophage isolate. However, it is worth noting that normalized recruited reads for all target phages seem to be lower in the Tara Ocean metagenome. This could be due to the huge data size incorporating metagenomic reads from various sites around the earth, where high relative abundances in some sites could be averaged out. For example, out of 42 metagenomes from Tara Ocean, over 60% (7185) of reads recruited to the S-SRP02 genome were from two metagenomes ERR599359 (Atlantic Ocean Near Joinville Island group, *N* = −62.0385, *E* = −49.529) and ERR599369 (Pacific Ocean near Peru, *N* = −5.2669, *E* = −85.2732), indicating the dominance of viruses similar to S-SRP02 from these two sites. The highest S-SRP02 relative abundance was observed in the Lake Baikal metagenome where S-SRP02 occurred at similar levels compared to major clusters of cyanopodovirus infecting *Synechococcus* spp. and *Prochlorococcus* spp. This indicates that S-SRP02-like phages are major players in the Lake Baikal virome.

### Coverage of S-SRP02 in Metagenome

Extensive mapping of viral metagenomic reads to S-SRP02 genes seems to suggest good coverage of S-SRP02 in datasets such as ERR599359. To quantify coverage of S-SRP02 in ERR599359, bowtie2 was used to do local alignment of the S-SRP02 nucleotide sequence with ERR599359 raw reads (Langmead and Salzberg, 2012). Out of 49,979,449 reads, only 46 raw reads were concordantly mapped to the S-SRP02 genome nucleotide sequence. This difficulty in aligning the S-SRP02 nucleotide sequence with environmental raw reads demonstrates highly diverse viral genes, which reflects the huge amount of viral dark matter existing despite substantial efforts to sequence environmental viromes over the years.

### Re-discovering S-SRP02 From Viral Metagenome

Extensive viral metagenome read mapping to S-SRP02 genes in ERR599359 and ERR599369 suggest the possible presence of S-SRP02 in those samples. In an effort to re-discover the S-SRP02 genome, we performed assembly using MetaSPAdes version 3.13.0 (Nurk et al., 2017). Contigs less than 30 kbp were removed. Two contigs sharing a significant number of genes with S-SRP02 were identified from the assembly of ERR599359 and they are denoted as S-SRP02-like viral contig 1 (SLVC-1) and SLVC-2, respectively. SLVC-1 has a genome size of 39,076 bp and shares 19 homologous genes with S-SRP02. SLVC-2 has a genome size of 31,399 bp and shares 23 homologous genes with S-SRP02 (Supplementary Table 4). Like the uncultured phage from fosmid metagenomics in the Mediterranean deep chlorophyll maximum sample, SLVC-1 and SLVC-2 share functional genes responsible for viral morphogenesis, structural protein, and transcriptional regulation with S-SRP02. This indicates the conservation of functional genes within the lineage of S-SRP02-like phages. Given that SLVC-1 and SLVC-2 do not cover 50% of S-SRP02 genes, our finding is insufficient to re-discover S-SRP02 in ERR599359. Nonetheless, SLVC-1 and SLVC-2 represents the presence of cyanophages sharing similar functional and structural genes with S-SRP02 in the Pacific Ocean and further expands the group of uncultured viruses belonging to the same evolutionary lineage with S-SRP02.

## Conclusion

This study presents the isolation and characterization of a novel freshwater cyanophage infecting *Synechococcus* sp. S-SRP02. It represents a previously unknown lineage of cyanophage and expands current knowledge on poorly characterized freshwater cyanophages. Viruses closely related to S-SRP02 have been identified in Mediterranean Sea viral fosmids. Further evidence from metagenomic mapping suggests that S-SRP02 and its related viruses are likely to play important ecological roles in the environment.

## Data Availability Statement

The datasets presented in this study can be found in online repositories. The names of the repository/repositories and accession number(s) can be found below: https://www.ncbi.nlm. nih.gov/, MW822601.

## Author Contributions

DZ contributed to the conceptualization, planning, experimentation, data analysis, and writing of the manuscript. YH contributed to reviewing, editing, funding acquisition, and supervision of the work. KG contributed to the conceptualization, reviewing, editing, supervision, and funding acquisition of the work. All authors contributed to the article and approved the submitted version.

## Conflict of Interest

The authors declare that the research was conducted in the absence of any commercial or financial relationships that could be construed as a potential conflict of interest.

## Publisher’s Note

All claims expressed in this article are solely those of the authors and do not necessarily represent those of their affiliated organizations, or those of the publisher, the editors and the reviewers. Any product that may be evaluated in this article, or claim that may be made by its manufacturer, is not guaranteed or endorsed by the publisher.

## Footnotes

1
https://www.genome.jp/virushostdb/
