# Supplementary material for: Genomic Characterization of a Novel Freshwater Cyanophage Reveals a New Lineage of Cyanopodovirus
Source: Front Microbiol. 2022 Jan 12;12:768868. doi: 10.3389/fmicb.2021.768868 (PMC8790148; doi:10.3389/fmicb.2021.768868)
Supplement: Supplementary file 1 [file Data_Sheet_1.docx]

Supplementary table S1Primer pair used for quantifying S-SRP02

| Target gene | Primer | Sequence 5’ to 3’ |
| --- | --- | --- |
| S-SRP02 major capsid protein | C1F | CAC TAC AGC CTG CGT TCT GA |
|  | C1R | CAC CCA GTT GGA TCA CCG AA |

Supplementary Table S2 list of viral metagenomes used for recruitment analysis.

| Metagenome | Data size (fasta file) in Gb | Accesion Number | Type | Reference |
| --- | --- | --- | --- | --- |
| Lake Baikal | 0.947 | SRR5936590 | Freshwater |  |
| Lake Bourget | 0.227 | ERR019478 | Freshwater | (Roux et al., 2012) |
| Lake Pavin | 0.202 | ERR019477 | Freshwater | (Roux et al., 2012) |
| Lake Neagh | 15.7 | SRR4449190 | Freshwater | (Arkhipova et al., 2018) |
|  |  | SRR4449292 | Freshwater |  |
|  |  | SRR4449268 |  |  |
|  |  | SRR4449256 |  |  |
|  |  | SRR4449244 |  |  |
|  |  | SRR4449250 |  |  |
|  |  | SRR4449240 |  |  |
|  |  | SRR4449225 |  |  |
|  |  | SRR4449214 |  |  |
|  |  | SRR4449206 |  |  |
|  |  | SRR4447083 |  |  |
| Hanriver | 24.7 | ERR1823950-ERR1823954 | Freshwater | (Moon et al., 2020) |
| Chattahoochee | 21.6 | SRR8075992 | Freshwater | (Ruiz-Perez, Tsementzi, Hatt, Sullivan, & Konstantinidis, 2019) |
|  |  | SRR8075980 |  |  |
|  |  | SRR8075978 |  |  |
|  |  | SRR8075991 |  |  |
| Tara Ocean DCM sample | 361.6 | ERR2752143 | Marine | (Brum et al., 2015) |
|  |  | ERR2752144 |  |  |
|  |  | ERR594352 |  |  |
|  |  | ERR594355 |  |  |
|  |  | ERR594357 |  |  |
|  |  | ERR594360 |  |  |
|  |  | ERR594363 |  |  |
|  |  | ERR594366 |  |  |
|  |  | ERR594367 |  |  |
|  |  | ERR594371 |  |  |
|  |  | ERR594372 |  |  |
|  |  | ERR594373 |  |  |
|  |  | ERR594375 |  |  |
|  |  | ERR594377 |  |  |
|  |  | ERR594379 |  |  |
|  |  | ERR594380 |  |  |
|  |  | ERR594381 |  |  |
|  |  | ERR594382 |  |  |
|  |  | ERR594383 |  |  |
|  |  | ERR594385 |  |  |
|  |  | ERR594386 |  |  |
|  |  | ERR594387 |  |  |
|  |  | ERR594389 |  |  |
|  |  | ERR594390 |  |  |
|  |  | ERR594394 |  |  |
|  |  | ERR594397 |  |  |
|  |  | ERR594399 |  |  |
|  |  | ERR594402 |  |  |
|  |  | ERR594405 |  |  |
|  |  | ERR594408 |  |  |
|  |  | ERR594409 |  |  |
|  |  | ERR594413 |  |  |
|  |  | ERR594414 |  |  |
|  |  | ERR594415 |  |  |
|  |  | ERR599340 |  |  |
|  |  | ERR599345 |  |  |
|  |  | ERR599349 |  |  |
|  |  | ERR599359 |  |  |
|  |  | ERR599362 |  |  |
|  |  | ERR599369 |  |  |
|  |  | ERR599374 |  |  |
|  |  | ERR599377 |  |  |
|  |  |  |  |  |
| Lake_Michigan | 23.4 | SRR1915829 | Freshwater | (Watkins et al., 2016) |
|  |  | SRR1915851 |  |  |
|  |  | SRR1974489 |  |  |
|  |  | SRR1974498 |  |  |
|  |  | SRR1974500 |  |  |
|  |  | SRR1974502 |  |  |
|  |  | SRR1974504 |  |  |
|  |  | SRR1974506 |  |  |
|  |  | SRR1974511 |  |  |
|  |  | SRR1974513 |  |  |
|  |  | SRR1974515 |  |  |
|  |  | SRR1974496 |  |  |
|  |  | SRR1974497 |  |  |
|  |  | SRR1974499 |  |  |
|  |  | SRR1974501 |  |  |
|  |  | SRR1974503 |  |  |
|  |  | SRR1974505 |  |  |
|  |  | SRR1974507 |  |  |
|  |  | SRR1974508 |  |  |
|  |  | SRR1974510 |  |  |
|  |  | SRR1974490 |  |  |
|  |  | SRR1974509 |  |  |
|  |  | SRR1974488 |  |  |
|  |  | SRR1974491 |  |  |
|  |  | SRR1974493 |  |  |
|  |  | SRR1974494 |  |  |
|  |  | SRR1974495 |  |  |
|  |  | SRR1974512 |  |  |
|  |  | SRR1974514 |  |  |
|  |  | SRR1974516 |  |  |
|  |  | SRR1974517 |  |  |
|  |  | SRR1296481 |  |  |
|  |  | SRR1301999 |  |  |
|  |  | SRR1302020 |  |  |
|  |  | SRR1302010 |  |  |
| Polar_lake | 35.9 | SRR9159689 | Freshwater | (Labbé, Girard, Vincent, & Culley, 2020) |
|  |  | SRR9159691 | Freshwater |  |
|  |  | SRR9159697 | Freshwater |  |
|  |  | SRR9159698 | Freshwater |  |
|  |  | SRR9159700 | Freshwater |  |
|  |  | SRR9159702 | Freshwater |  |
|  |  | SRR9159688 | Freshwater |  |
|  |  | SRR9159690 | Freshwater |  |
|  |  | SRR9159692 | Freshwater |  |
|  |  | SRR9159695 | Freshwater |  |
|  |  | SRR9159699 | Freshwater |  |
|  |  | SRR9159701 | Freshwater |  |
|  |  | SRR9159693 | Freshwater |  |
|  |  | SRR9159694 | Freshwater |  |
|  |  | SRR9159696 | Freshwater |  |

Supplementary Table S3 genes sharing homology with S-SRP02 from uncultured phage from Mediterranean Sea Deep Chlorophyll Maximum viral fosmid

| Protein id | Putative function | % identity | Alignment length | evalue |
| --- | --- | --- | --- | --- |
| YP_009777923.1 | terminase large subunit [uncultured phage_MedDCM-OCT-S38-C3] | 64.605 | 582 | 0 |
| YP_009777942.1 | putative tail tubular protein B [uncultured phage_MedDCM-OCT-S38-C3] | 57.342 | 647 | 0 |
| YP_009777938.1 | head-tail connector protein [uncultured phage_MedDCM-OCT-S38-C3] | 58.452 | 491 | 1.53E-175 |
| YP_009777926.1 | DNA-directed RNA polymerase [uncultured phage_MedDCM-OCT-S38-C3] | 40.206 | 776 | 3.22E-162 |
| YP_009777947.1 | hypothetical protein HOQ56_gp25 [uncultured phage_MedDCM-OCT-S38-C3] | 32.322 | 1154 | 1.87E-158 |
| YP_009777940.1 | minor capsid protein 10 [uncultured phage_MedDCM-OCT-S38-C3] | 53.107 | 354 | 3.37E-108 |
| YP_009777946.1 | hypothetical protein HOQ56_gp24 [uncultured phage_MedDCM-OCT-S38-C3] | 31.437 | 1037 | 2.58E-90 |
| YP_009777944.1 | hypothetical protein HOQ56_gp22 [uncultured phage_MedDCM-OCT-S38-C3] | 55.462 | 238 | 1.12E-75 |
| YP_009777941.1 | tail tubular protein A [uncultured phage_MedDCM-OCT-S38-C3] | 55.665 | 203 | 1.13E-66 |
| YP_009777945.1 | hypothetical protein HOQ56_gp23 [uncultured phage_MedDCM-OCT-S38-C3] | 36.062 | 452 | 4.61E-64 |
| YP_009777934.1 | 5'-3' exonuclease (including N-terminal domain of PolI) (Exo) [uncultured phage_MedDCM-OCT-S38-C3] | 43.145 | 248 | 5.20E-59 |
| YP_009777955.1 | Integrase (XerC) [uncultured phage_MedDCM-OCT-S38-C3] | 37.943 | 282 | 1.36E-52 |
| YP_009777930.1 | hypothetical protein HOQ56_gp08 [uncultured phage_MedDCM-OCT-S38-C3] | 55.556 | 162 | 1.75E-51 |
| YP_009777929.1 | transcriptional regulator NrdR [uncultured phage_MedDCM-OCT-S38-C3] | 44.103 | 195 | 1.37E-48 |
| YP_009777939.1 | capsid assembly protein [uncultured phage_MedDCM-OCT-S38-C3] | 40.816 | 245 | 1.13E-46 |
| YP_009777956.1 | Transcriptional regulators (MarR) [uncultured phage_MedDCM-OCT-S38-C3] | 57.547 | 106 | 3.53E-35 |
| YP_009777928.1 | hypothetical protein HOQ56_gp06 [uncultured phage_MedDCM-OCT-S38-C3] | 47.2 | 125 | 2.21E-31 |
| YP_009777924.1 | hypothetical protein HOQ56_gp02 [uncultured phage_MedDCM-OCT-S38-C3] | 58.947 | 95 | 2.55E-30 |
| YP_009777935.1 | hypothetical protein HOQ56_gp13 [uncultured phage_MedDCM-OCT-S38-C3] | 45.865 | 133 | 4.07E-26 |
| YP_009777927.1 | hypothetical protein HOQ56_gp05 [uncultured phage_MedDCM-OCT-S38-C3] | 37.297 | 185 | 2.97E-25 |
| YP_009777931.1 | hypothetical protein HOQ56_gp09 [uncultured phage_MedDCM-OCT-S38-C3] | 56.923 | 65 | 2.50E-21 |
| YP_009777943.1 | putative acetyltransferase [uncultured phage_MedDCM-OCT-S38-C3] | 36.184 | 152 | 1.20E-19 |
| YP_009777948.1 | phage related tail fibre protein [uncultured phage_MedDCM-OCT-S38-C3] | 35.135 | 111 | 2.97E-18 |
| YP_009777957.1 | hypothetical protein HOQ56_gp35 [uncultured phage_MedDCM-OCT-S38-C3] | 35.417 | 96 | 4.95E-18 |
| YP_009777896.1 | putative tail tubular protein B [uncultured phage_MedDCM-OCT-S31-C1] | 57.259 | 861 | 0 |
| YP_009777906.1 | terminase large subunit [uncultured phage_MedDCM-OCT-S31-C1] | 57.877 | 584 | 0 |
| YP_009777900.1 | head-tail connector protein [uncultured phage_MedDCM-OCT-S31-C1] | 56.126 | 506 | 0 |
| YP_009777920.1 | Mitochondrial DNA-directed RNA polymerase (RPO41) [uncultured phage_MedDCM-OCT-S31-C1] | 34.833 | 778 | 5.55E-119 |
| YP_009777898.1 | major capsid protein [uncultured phage_MedDCM-OCT-S31-C1] | 55.362 | 345 | 1.25E-115 |
| YP_009777897.1 | tail tubular protein A [uncultured phage_MedDCM-OCT-S31-C1] | 59.624 | 213 | 1.04E-81 |
| YP_009777884.1 | exonuclease [uncultured phage_MedDCM-OCT-S31-C1] | 44.747 | 257 | 4.35E-68 |
| YP_009777894.1 | hypothetical protein HOQ55_gp15 [uncultured phage_MedDCM-OCT-S31-C1] | 44.444 | 216 | 8.12E-50 |
| YP_009777899.1 | capsid assembly protein [uncultured phage_MedDCM-OCT-S31-C1] | 40.083 | 242 | 5.65E-43 |
| YP_009777919.1 | integrase family protein [uncultured phage_MedDCM-OCT-S31-C1] | 30.12 | 332 | 6.84E-43 |
| YP_009777918.1 | Transcriptional regulators (MarR) [uncultured phage_MedDCM-OCT-S31-C1] | 52.778 | 108 | 1.80E-26 |
| YP_009777895.1 | gp42 [uncultured phage_MedDCM-OCT-S31-C1] | 37.748 | 151 | 4.58E-24 |
| YP_009777890.1 | phage tail fiber protein [uncultured phage_MedDCM-OCT-S31-C1] | 40.496 | 121 | 5.88E-24 |
| YP_009777922.1 | hypothetical protein HOQ55_gp41 [uncultured phage_MedDCM-OCT-S31-C1] | 42.017 | 119 | 3.27E-20 |
| YP_009777921.1 | hypothetical protein HOQ55_gp42 [uncultured phage_MedDCM-OCT-S31-C1] | 33.333 | 183 | 3.03E-18 |
| YP_009777903.1 | hypothetical protein HOQ55_gp24 [uncultured phage_MedDCM-OCT-S31-C1] | 38.4 | 125 | 1.14E-12 |
| YP_009777893.1 | hypothetical protein HOQ55_gp14 [uncultured phage_MedDCM-OCT-S31-C1] | 27.897 | 233 | 3.76E-11 |
| YP_009777880.1 | transcriptional regulator NrdR [uncultured phage_MedDCM-OCT-S31-C1] | 45.833 | 48 | 2.51E-08 |
| YP_009777629.1 | terminase large subunit [uncultured phage_MedDCM-OCT-S37-C6] | 58.562 | 584 | 0 |
| YP_009777598.1 | hypothetical protein HOQ49_gp05 [uncultured phage_MedDCM-OCT-S37-C6] | 34.123 | 1140 | 0 |
| YP_009777635.1 | head-tail connector protein [uncultured phage_MedDCM-OCT-S37-C6] | 59.316 | 526 | 0 |
| YP_009777597.1 | chromosome segregation ATPase-like protein [uncultured phage_MedDCM-OCT-S37-C6] | 39.639 | 830 | 6.34E-150 |
| YP_009777618.1 | DNA-directed RNA polymerase [uncultured phage_MedDCM-OCT-S37-C6] | 34.833 | 778 | 1.78E-126 |
| YP_009777637.1 | major capsid protein [uncultured phage_MedDCM-OCT-S37-C6] | 54.023 | 348 | 1.66E-105 |
| YP_009777638.1 | tail tubular protein A [uncultured phage_MedDCM-OCT-S37-C6] | 64.623 | 212 | 2.82E-94 |
| YP_009777611.1 | DNA polymerase I (TIGR00593) [uncultured phage_MedDCM-OCT-S37-C6] | 48.77 | 244 | 8.15E-75 |
| YP_009777595.1 | hypothetical protein HOQ49_gp02 [uncultured phage_MedDCM-OCT-S37-C6] | 59.144 | 257 | 1.03E-72 |
| YP_009777596.1 | hypothetical protein HOQ49_gp03 [uncultured phage_MedDCM-OCT-S37-C6] | 34.029 | 479 | 2.57E-61 |
| YP_009777636.1 | capsid assembly protein [uncultured phage_MedDCM-OCT-S37-C6] | 39.916 | 238 | 2.33E-44 |
| YP_009777599.1 | hypothetical protein HOQ49_gp06 [uncultured phage_MedDCM-OCT-S37-C6] | 47.541 | 122 | 1.12E-30 |
| YP_009777594.1 | putative acetyltransferase [uncultured phage_MedDCM-OCT-S37-C6] | 43.333 | 150 | 6.93E-30 |
| YP_009777620.1 | Transcriptional regulators (MarR) [uncultured phage_MedDCM-OCT-S37-C6] | 42.735 | 117 | 1.17E-26 |
| YP_009777632.1 | hypothetical protein HOQ49_gp39 [uncultured phage_MedDCM-OCT-S37-C6] | 43.182 | 132 | 5.28E-26 |
| YP_009777617.1 | phage single-stranded DNA-binding protein [uncultured phage_MedDCM-OCT-S37-C6] | 34.146 | 164 | 3.74E-21 |
| YP_009777633.1 | hypothetical protein HOQ49_gp40 [uncultured phage_MedDCM-OCT-S37-C6] | 50 | 114 | 1.68E-20 |
| YP_009777616.1 | Holliday junction resolvase (Rus) [uncultured phage_MedDCM-OCT-S37-C6] | 39.785 | 93 | 1.69E-17 |
| YP_009777630.1 | hypothetical protein HOQ49_gp37 [uncultured phage_MedDCM-OCT-S37-C6] | 36.471 | 85 | 5.86E-08 |

Supplementary Table S4 genes sharing homology with S-SRP02 from SLVC assembled from Tara Ocean DCM sample ERR599359

| Putative function | % Identity | E-value | Homologous with |  |
| --- | --- | --- | --- | --- |
| C39 family peptidase | 34 | 6.84E-07 | S-SRP02_10 | SLVC-1 |
| hypothetical protein | 59 | 0 | S-SRP02_11 |  |
| hypothetical protein | 48 | 1.59E-30 | S-SRP02_19 |  |
| MarR family transcriptional regulator | 32 | 3.12E-37 | S-SRP02_20 |  |
| hypothetical protein | 32 | 5.68E-136 | S-SRP02_24 |  |
| DNA-directed RNA polymerase | 32 | 2.75E-20 | S-SRP02_25 |  |
| phage single-stranded DNA-binding protein | 41 | 1.91E-26 | S-SRP02_26 |  |
| RusA family crossover junction endodeoxyribonuclease | 42 | 1.64E-11 | S-SRP02_27 |  |
| hypothetical protein | 47 | 1.78E-78 | S-SRP02_33 |  |
| hypothetical protein | 47 | 3.15E-33 | S-SRP02_35 |  |
| hypothetical protein | 46 | 8.03E-26 | S-SRP02_36 |  |
| hypothetical protein | 59 | 0 | S-SRP02_37 |  |
| head-to-tail connector(portal) protein | 32 | 1.46E-31 | S-SRP02_38 |  |
| capsid assembly protein | 53 | 8.25E-112 | S-SRP02_39 |  |
| major capsid protein | 50 | 6.01E-76 | S-SRP02_40 |  |
| tail tubular protein A | 69 | 0 | S-SRP02_41 |  |
| tail tubular protein B | 44 | 9.83E-28 | S-SRP02_42 |  |
| putative acetyltransferase | 52 | 6.38E-72 | S-SRP02_43 |  |
| hypothetical protein | 33 | 4.50E-17 | S-SRP02_44 |  |
| hypothetical protein | 42 | 1.37E-24 | S-SRP02_18 | SLVC-2 |
| hypothetical protein | 57 | 5.23E-39 | S-SRP02_19 |  |
| MarR family transcriptional regulator | 34 | 5.23E-61 | S-SRP02_20 |  |
| hypothetical protein | 52 | 0 | S-SRP02_24 |  |
| DNA-directed RNA polymerase | 36 | 1.34E-24 | S-SRP02_25 |  |
| RusA family crossover junction endodeoxyribonuclease | 45 | 4.67E-50 | S-SRP02_27 |  |
| transcriptional regulator NrdR | 57 | 1.31E-47 | S-SRP02_28 |  |
| hypothetical protein | 50 | 1.74E-80 | S-SRP02_33 |  |
| DNA polymerase I | 63 | 2.12E-07 | S-SRP02_34 |  |
| hypothetical protein | 56 | 6.74E-46 | S-SRP02_35 |  |
| hypothetical protein | 47 | 2.83E-36 | S-SRP02_36 |  |
| hypothetical protein | 58 | 0 | S-SRP02_37 |  |
| head-to-tail connector(portal) protein | 36 | 4.80E-44 | S-SRP02_38 |  |
| capsid assembly protein | 79 | 0 | S-SRP02_39 |  |
| major capsid protein | 75 | 7.25E-122 | S-SRP02_40 |  |
| tail tubular protein A | 75 | 0 | S-SRP02_41 |  |
| tail tubular protein B | 60 | 1.16E-55 | S-SRP02_42 |  |
| putative acetyltransferase | 69 | 5.04E-118 | S-SRP02_43 |  |
| hypothetical protein | 32 | 6.51E-103 | S-SRP02_44 |  |
| N-acetylmuramoyl-L-alanine amidase | 38 | 2.21E-67 | S-SRP02_45 |  |
| chromosome segregation ATPase-like protein | 30 | 2.90E-133 | S-SRP02_46 |  |
| hypothetical protein | 34 | 3.61E-16 | S-SRP02_47 |  |

Arkhipova, K., Skvortsov, T., Quinn, J. P., McGrath, J. W., Allen, C. C. R., Dutilh, B. E., . . . Kulakov, L. A. (2018). Temporal dynamics of uncultured viruses: a new dimension in viral diversity. *The ISME Journal, 12*(1), 199-211. doi:10.1038/ismej.2017.157

Brum, J. R., Ignacio-Espinoza, J. C., Roux, S., Doulcier, G., Acinas, S. G., Alberti, A., . . . Sullivan, M. B. (2015). Patterns and ecological drivers of ocean viral communities. *Science, 348*(6237), 1261498. doi:10.1126/science.1261498

Labbé, M., Girard, C., Vincent, W. F., & Culley, A. I. (2020). Extreme Viral Partitioning in a Marine-Derived High Arctic Lake. *mSphere, 5*(3), e00334-00320. doi:10.1128/mSphere.00334-20

Moon, K., Jeon, J. H., Kang, I., Park, K. S., Lee, K., Cha, C.-J., . . . Cho, J.-C. (2020). Freshwater viral metagenome reveals novel and functional phage-borne antibiotic resistance genes. *Microbiome, 8*(1), 75-75. doi:10.1186/s40168-020-00863-4

Roux, S., Enault, F., Robin, A., Ravet, V., Personnic, S., Theil, S., . . . Debroas, D. (2012). Assessing the diversity and specificity of two freshwater viral communities through metagenomics. *PLoS One, 7*(3), e33641-e33641. doi:10.1371/journal.pone.0033641

Ruiz-Perez, C. A., Tsementzi, D., Hatt, J. K., Sullivan, M. B., & Konstantinidis, K. T. (2019). Prevalence of viral photosynthesis genes along a freshwater to saltwater transect in Southeast USA. *Environ Microbiol Rep, 11*(5), 672-689. doi:10.1111/1758-2229.12780

Watkins, S. C., Kuehnle, N., Ruggeri, C. A., Malki, K., Bruder, K., Elayyan, J., . . . Putonti, C. (2016). Assessment of a metaviromic dataset generated from nearshore Lake Michigan. *Marine and Freshwater Research, 67*(11), 1700-1708. doi:org/10.1071/MF15172
